# Ultrahigh Sensitivity Mach−Zehnder Interferometer Sensor Based on a Weak One-Dimensional Field Confinement Silica Waveguide

**DOI:** 10.3390/s21196600

**Published:** 2021-10-02

**Authors:** Chenming Zhao, Lei Xu, Liying Liu

**Affiliations:** Key Lab for Micro and Nanophotonic Structures (Ministry of Education) of Ultra Precision Optical Manufacturing, Shanghai Engineering Research Center, Department of Optical Science and Engineering, Fudan University, Shanghai 200433, China; chenmingzhao13@fudan.edu.cn (C.Z.); lei_xu@fudan.edu.cn (L.X.)

**Keywords:** Mach−Zehnder interferometer sensor, weak confinement waveguide, waveguide sensitivity

## Abstract

We report a novel Mach−Zehnder interferometer (MZI) sensor that utilizes a weak one-dimensional field confinement silica waveguide (WCSW). The WCSW has a large horizontal and vertical aspect ratio and low refractive index difference, which features easy preparation and a large evanescent field for achieving high waveguide sensitivity. We experimentally achieved WCSW ultrahigh waveguide sensitivity of 0.94, MZI sensitivity of 44,364 π/RIU and a low limit of detection (*LOD*) of 6.1 × 10^−7^ RIU.

## 1. Introduction

Integrated optical sensors [1,2] have developed rapidly in recent years due to their significant advantages, such as compactness, stability, capability of integration and high level of sensitivity. They have already been widely applied in areas such as food security, biological detection, medical hygiene and environmental monitoring. Recently, various types of ultrahigh sensitivity refractive index optical sensors with a sensitivity of 10^3^–10^6^ nm/RIU have been proposed, including plasmonic waveguides [3], Mach−Zehnder interferometers (MZI) [4,5,6] and ring resonators [7]. Among these configurations, MZI sensors feature easy fabrication, a long interaction length and the ability of phase measurement. Optical waveguides are widely adopted as a core sensing platform due to their mechanical stability, miniaturization, ability to be mass produced and immunity to electromagnetic interference. The sensitivity of a waveguide sensor is determined by both waveguide sensitivity (*S_w_*) and device sensitivity (*S_d_*) [8]. Device sensitivity depends on the configuration of the optical sensor with the units of nm/RIU, dB/RIU or rad/RIU when wavelength, intensity and phase-based interrogation are used, respectively. Waveguide sensors mainly rely on the evanescent field for sensing, so waveguide sensitivity depends on the overlap of evanescent field with the analytes. Although the limit of *S_w_* is 1, this value is much lower in conventional waveguides because a large part of the optical field confines practically inside the waveguide rather than as an evanescent field (tail of evanescent field in the order of 100 nm). Evanescent field strength is directly dependent on the cross-sectional dimensions of the waveguide and the refractive indexes of the waveguide/analytes. Thus, the most significant waveguide sensitivity gain is expected from optimizing the waveguide structure for a large evanescent field. Lots of work has been proposed by enlarging the evanescent field to increase *S_w_* [9,10,11,12,13]. For instance, the TM mode in silicon photonic wire shows a higher waveguide sensitivity of 0.35 because most of the field intensity is above and beneath the waveguide core, offering a strong field−matter interaction [9]. The evanescent field in the reverse symmetry waveguide has deeper penetration into the upper cladding to provide higher sensing sensitivity, thus, a large *S_w_* of 0.58 was obtained [10]. By inserting a metal layer between the substrate and waveguide (metal clad leaky waveguide, MCLW), the evanescent field expands, a high *S_w_* of 0.544 is expected [11]. Slot waveguides have enhanced the evanescent field in the low refractive index slot, which enables the optical field to contact with analytes more efficiently and *S_w_* increases to 0.41, as estimated from [12]. However, all these waveguide structure add complexity and cost to the technique for mass production.

In this paper, we report on a high waveguide sensitivity close to 1 by utilizing a new waveguide structure, i.e., weak one-dimensional field confinement silica waveguide (WCSW) which has a large horizontal and vertical aspect ratio and low refractive index difference [14,15]. The WCSW is easy to prepare due to the large width and shows low sidewall loss because the waveguide material has a low refractive index difference. We also prepared MZI waveguide sensors with WCSW, combining the advantages of the MZI sensor and the WCSW, ultrahigh device sensitivity of 44,364 π/RIU and a low limit of detection (*LOD*) of 6.1 × 10^−7^ RIU were achieved for refractive index sensing.

## 2. Waveguide Design and Simulations

**Figure 1:**
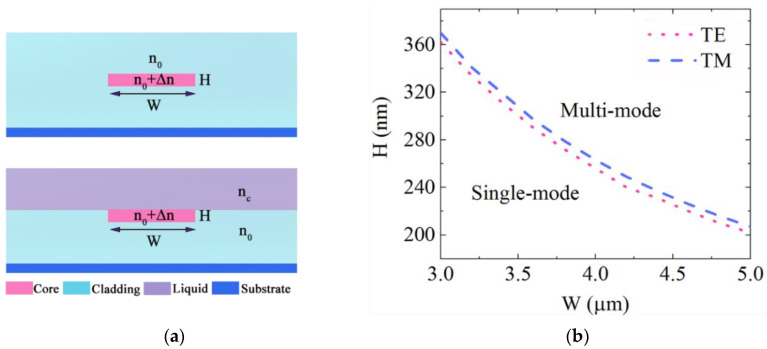
(**a**) Cross section of the WCSW with width *W* and height *H* with upper cladding (up) and sensing liquid (down). The refractive indices of claddings, core and liquid are *n*_0_, *n*_0_ + ∆*n* and *n_c_*, respectively. (**b**) Single-mode condition for TE and TM polarizations of WCSW at *n*_0_ = 1.466 and ∆*n* = 0.03.

Figure 1a shows schematically the WCSW structure. It has a large horizontal and vertical aspect ratio and low refractive index difference. The optical field is confined strongly inside the waveguide core in the horizontal direction but weakly in the vertical direction, therefore the evanescent field is largely distributed in the claddings. The properties of the WCSW have already been analyzed and details can be found in our previous work [15]. Thus, we will focus on the sensing behavior of WCSW in this paper. The waveguide is designed with a width (*W*) of 4 μm and a thickness (*H*) of 250 nm. The refractive indices of the cladding and core layer are *n*_0_ = 1.466 and *n*_1_ = *n*_0_ + ∆*n* = 1.496, respectively. We used the finite difference beam propagation method (FD-BPM) via BeamPROP software (RSoft Design Group, Inc.) to simulate the waveguide properties. At λ = 632.8 nm the single-mode region at ∆*n* = 0.03 is illustrated in Figure 1b for both TE and TM polarizations. This figure shows that the single-mode cutoff thickness is 263 nm at waveguide width 4 μm for TM polarization thus only a single mode propagates in the waveguide of 250 nm thickness.

**Figure 2:**
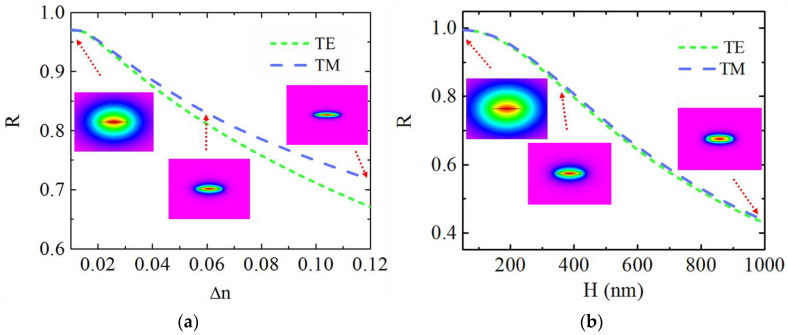
The evanescent field ratio *R* as functions of (**a**) ∆*n* at *H* = 250 nm and (**b**) *H* at ∆*n* = 0.03 for TE and TM fundamental modes. Insets: TM fundamental mode fields when (**a**) ∆*n* = 0.01, 0.06, 0.12 and (**b**) *H* = 50 nm, 350 nm, 1000 nm, respectively.

The optical field distributed in the claddings of the WCSW can be influenced notably by the waveguide parameters. The evanescent field ratio (EFR), the ratio of the optical field intensity in the claddings to the total mode field intensity, can be defined as:

(1) $$R=\frac{I_{claddings}}{I_{\infty }}=\frac{\iint_{claddings}{\left|E(x,y)\right|}^{2}dxdy}{\iint_{\infty }{\left|E(x,y)\right|}^{2}dxdy}$$

where *I_claddings_* and *I_∞_* denote the intensities of the optical field distributed in the claddings and the total mode field, respectively, **E**(*x*,*y*) is the electric field. Figure 2a shows the evanescent field ratio *R* as a function of the refractive index difference ∆*n* (@*W* = 4 μm, *H* = 250 nm) for TE and TM fundamental modes, respectively. The insets exhibit the TM fundamental mode fields when ∆*n* = 0.01, 0.06, 0.12. The confinement of the mode field enhances as ∆*n* increases, resulting in a decreased ratio of mode field penetrating into the cladding and *R* reduces. The *R* of TM mode is always larger than that of TE mode, this is a result of electric field discontinuity on the boundary of claddings/core. When is ∆*n* small, the *R* divergency of TE and TM modes becomes small due to the low electric field discontinuity (more details in [15]). *R* can reach up to 97.0% when ∆*n* = 0.01, which indicates that most of the optical field leaks to the cladding. Figure 2b shows the evanescent field ratio *R* as a function of *H* (@*W* = 4 μm, ∆*n* = 0.03) for TE and TM fundamental modes, respectively. The insets are the TM fundamental mode fields when *H* = 50 nm, 350 nm, 1000 nm, respectively. As *H* changes from 50 nm to 1000 nm, *R* gradually reduces because of the enhanced confinement for both TE and TM fundamental modes. *R* can reach as large as 99.5% at *H* = 50 nm. As a comparison, the polymer waveguide in [16] has an evanescent field ratio of 10.8%, at most. The hybrid plasmonic waveguide in [17] confines ~60% and ∼82% of the evanescent field in the dielectric slot and an active sensing region. The electric field discontinuity still introduces a larger *R* of TM mode than TE mode, but the distinction is not large between them as revealed in Figure 2b due to the small ∆*n*. As a result, we can conclude that *R* of WCSW is insensitive to TE/TM polarizations with small ∆*n*. WCSW has large evanescent field even though its lateral dimension is several micrometers, which has potential for high waveguide sensitivity sensing at low manufacture cost.

**Figure 3:**
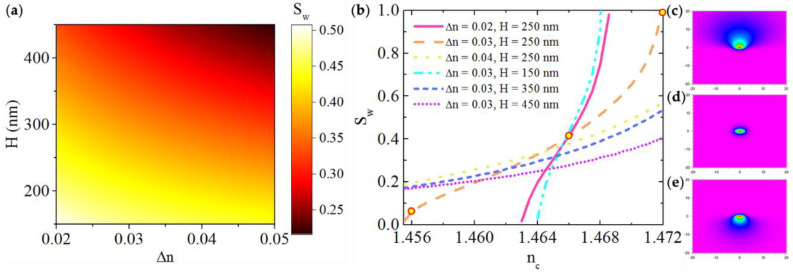
(**a**) Variation of waveguide sensitivity *S_w_* (TM fundamental mode) with different ∆*n* and *H* with symmetric field distribution *n_c_* = *n*_0_. (**b**) Variation of waveguide sensitivity *S_w_* (TM fundamental mode) with different ∆*n* and *H* when *n_c_* changes from 1.456 to 1.472. (**c**–**e**) The fundamental mode distributions of *n_c_* = 1.472, *n_c_* = 1.466 and *n_c_* = 1.456 at ∆*n* = 0.03, *H* = 250 nm, respectively.

The waveguide sensitivity *S_w_* can be described as [18]:

(2) $$S_{w}=\frac{\partial N_{eff}}{\partial n_{c}}$$

where *N_eff_* is the mode effective refractive index, *n_c_* is the refractive index of the upper cladding (the sensing liquid). According to [18], the waveguide sensitivity *S_w_* for TM mode in planar waveguide can be expressed as:

(3) $$S_{w}=\frac{\partial N_{eff}}{\partial n_{c}}=\frac{n_{c}R}{N_{eff}}\left[2{\left(\frac{N_{eff}}{n_{c}}\right)}^{2}-1\right]$$

while for a strip waveguide, the exact analytical solution is hard to derive. Nevertheless, the concept that *S_w_* depends proportionally on the evanescent field ratio *R* is commonly acceptable [19]. Considering the symmetric field distribution of the waveguide in Figure 1a, where *n_c_* = *n*_0_, only half of the evanescent field overlaps with the liquid in the upper cladding. Thus *R* is supposed to be limited <50% and leads to *S_w_* less than 0.5. *S_w_* for the TM fundamental mode is calculated as a function of *H* and ∆*n* by BeamPROP and showed in Figure 3a. The trends of *S_w_* and *R* versus *H* and ∆*n* are alike where smaller *H* and ∆*n* cause higher *S_w_*. If *n_c_* > *n*_0_, the optical field is asymmetrically distributed with a higher *R* and the waveguide sensitivity can be further increased. Figure 3b shows the variation of *S_w_* as a function of *n_c_* at different *H* and ∆*n* combinations. The TM fundamental mode field distributions of *n_c_* = 1.472, 1.466 and 1.456 at ∆*n* = 0.03, *H* = 250 nm are shown in Figure 3c, d and e respectively. The results show that when *n_c_* increases, the mode field is pulled upward to the cladding, therefore the evanescent field interacts more with the liquid and *S_w_* increases as *n_c_* grows. This is the case of a reverse symmetry waveguide (*n_c_* > *n*_0_) [10]. When *n_c_* increases to the mode cutoff value, *S_w_* approaches 1. This can be explained thus: when a larger portion of evanescent field penetrates into the upper cladding, *N_eff_* approaches *n_c_*. When *H* or ∆*n* is fixed, the sensitivity curves become steeper with lower ∆*n* or *H*, respectively. This is because for a lower ∆*n* or *H*, the evanescent field is larger, thus more likely disturbed by *n_c_*. Among all the calculated cases, parameter combinations of ∆*n* = 0.02, *H* = 250 nm; ∆*n* = 0.03, *H* = 250 nm and ∆*n* = 0.03, *H* = 150 nm exhibited a waveguide sensitivity close to 1, meanwhile the waveguide still maintained single mode operation. As ∆*n* = 0.03, *H* = 250 nm combination had a wide refractive index sensing range from less than 1.456 to 1.472, it was chosen for sample preparation.

## 3. MZI Sensitivity

**Figure 4:**
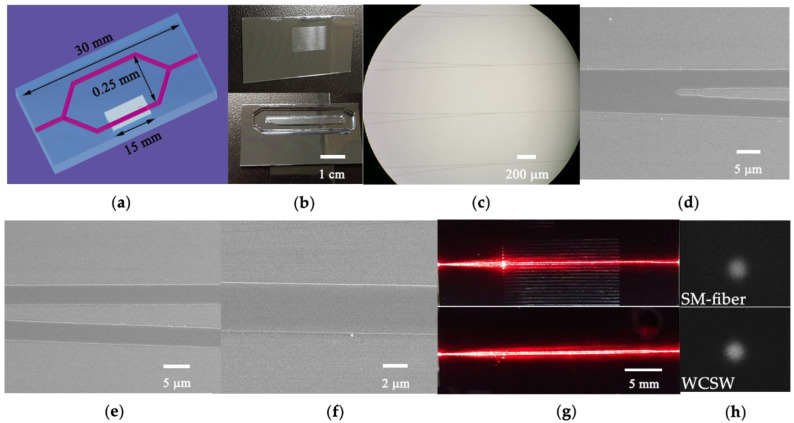
(**a**) Schematic diagram of the MZI sensor. (**b**) The fabricated MZI sensors before and after sealing with PDMS channel. (**c**) Photograph of S-bend splitters. (**d**–**f**) SEM images of S-bend, two arms and zoomed one arm of the MZI. (**g**) Light propagation when sensing arm in air (upper image) and DMSO/solutions (lower image). (**h**) Near field spots of the fiber mode and TM fundamental mode of WCSW.

In an MZI type sensor (Figure 4a), the input light is split into two beams at the first splitter and one is traveling through the reference arm while the other is traveling through the sensing arm. Interaction between the analytes and the optical field will induce an extra phase ∆*ϕ* in the sensing arm. The MZI sensitivity (*S_d_*), defined as the phase difference between the two arms changing with the analyte refractive index, can be described as:

(4) $$S_{d}=\frac{\partial \phi }{\partial n_{c}}=\frac{\partial \phi }{\partial N_{eff}}\frac{\partial N_{eff}}{\partial n_{c}}=\frac{2\pi }{\lambda }LS_{w}$$

where *L* is the interaction length in the sensing area and *λ* is the wavelength. The phase difference is then written as:

(5) $$\Delta \phi =S_{d}\Delta n_{c}$$

Light beams in two arms recombine at the second splitter and interference occurs due to the phase difference ∆*ϕ*. The output intensity *I_out_*, which is a periodic function of ∆*ϕ*, can be written as [12]:

(6) $$I_{out}\propto I_{in}[1+V\cos(\Delta \phi +\phi_{0})]$$

where *I_in_* is the input light intensity, *ϕ*_0_ is intrinsic phase difference induced by the asymmetric of two arms, *V* is the extinction ratio with the range 0 to 1.

∆*ϕ* can be measured from the output intensity, as shown in Equation (6). Equations (4) and (5) reveal that measured phase difference ∆*ϕ* is proportional to the waveguide sensitivity *S_w_*, sensing area length *L* and reversely proportional to *λ*. Therefore, by optimizing the waveguide sensitivity and device configuration, the best performance condition of the sensing device can be determined.

## 4. Waveguide and MZI Fabrication

The MZI sensors based on WCSW were fabricated on Si wafers by sol−gel technique [20] and dip-coating and rapid thermal annealing (DC-RTA) [21].The silica substrate layer with a thickness of 8 μm was prepared with tetraethoxysilane (TEOS). The core layer, Zr^4+^ doped silica, was prepared on the silica substrate from a sol composed of methacryloxypropyl trimethoxysilane (MAPTMS), zirconium n-propoxide (ZPO) and methacrylic acid (MAA). Sample preparation details can be found in our previous work in [15]. The MZI pattern was formed on the chip after processes of UV photolithography (Karl Suss MJB3), development (RZJ-304) and wet etching (BOE solution). The upper cladding with a thickness of 8 μm was then fabricated to isolate the waveguide core from environment. Sensing windows were fabricated on the upper silica cladding by alignment photolithography and wet etching to expose the surface of sensing arm in its surroundings. The whole chip was then annealed at 1000 °C for 1 h to densify the films. Finally, a polydimethylsiloxane (PDMS) channel was fabricated on the chip for liquid delivery. The MZI sensors on the Si wafer were robust, reusable and nonreactive to most of the measured liquids.

The fabricated waveguides were characterized through the prism coupling device and surface profiler (Zygo NV200), the parameters of core refractive index *n*_1_ = 1.496, claddings refractive index *n*_0_ = 1.466, core layer thickness *H* = 250 ± 10 nm and width *W* = 4 μm were obtained. The total length and interaction area length of the MZI sensors were 30 mm and 15 mm, as illustrated in Figure 4a. The images of the fabricated MZI sensor arrays before and after sealing with PDMS channel are shown in Figure 4b. The adjacent MZI sensors were 500 μm apart, which was large enough to avoid disturbance with each other. S-bend splitters with a curvature radius of 25 mm were designed for a lower splitting loss (as shown in Figure 4c). Figure 4d–f show SEM images of S-bend splitter, sensing and reference arms and zoomed one arm of fabricated waveguide before adding the upper cladding, respectively. The smooth surface of the waveguides prepared by sol−gel technique was observed in these figures. Figure 4g shows images when the sensing arm is in air (upper image) and in DMSO/water solution (lower image). When the sensing arm was exposed in air, the propagating mode was cutoff, thus light was terminated although a part of light was still scattered into the sensing area. With DMSO/water solution filled in the sensing arm, fundamental mode was supported and light propagated, as shown in Figure 4g (lower image).

## 5. Experimental Results and Discussion

**Figure 5:**
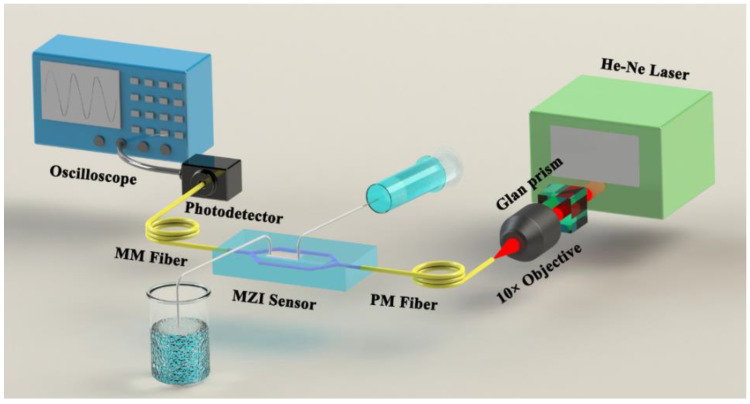
Schematic diagram of the MZI sensor measurement setup. PM fiber: polarization maintaining fiber, MM fiber: multi-mode fiber.

Figure 5 shows the schematic diagram of the measurement setup. A 632.8 nm He-Ne laser was polarized by a Glan prism and coupled into a polarization maintaining (PM) single-mode optical fiber through a 10× objective. TM polarized light from the single-mode fiber was then end-fire coupled into the straight waveguide of MZI sensor from the edge of the chip. A 5D alignment stage controlled by a piezo stepper actuator was used to align the fiber and waveguide for high efficiency coupling. Light coming out of the MZI was collected by a multimode (MM) optical fiber. After detection by a photodetector, the signals were shown on an oscilloscope (Tektronix TDS3012C). By measuring the attenuation of the propagation line intensity of a 1.9 cm long straight waveguide, low propagation loss of 1.24 dB/cm at 632.8 nm was obtained. The low propagation loss benefits from the low absorption of waveguide material silica and uniform film prepared by sol−gel technique and DC-RTA. The near-field spots of fiber mode and TM fundamental mode of WCSW were imaged by a visible light CCD camera and shown in Figure 4h, the single-mode property of WCSW was confirmed. The insertion loss of the straight waveguide was 3.28 dB and the coupling loss of the WCSW with single-mode fiber was 0.93 dB. Low coupling loss results from the high overlap of the waveguide mode and fiber mode as shown in Figure 4h. By measuring the input light intensity and the MZI output interference maximum, the insertion loss of the MZI sensor was obtained to be 7.89 dB.

**Figure 6:**
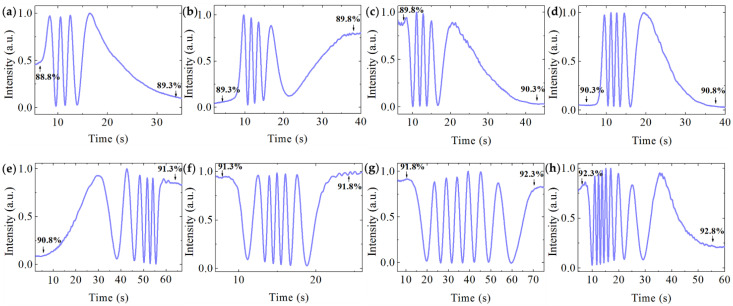
Interferograms of the MZI sensor measuring different volume percentage DMSO/water solutions. (**a**) 88.8% → 89.3%, (**b**) 89.3% → 89.8%, (**c**) 89.8% → 90.3%, (**d**) 90.3% → 90.8%, (**e**) 90.8% → 91.3%, (**f**) 91.3% → 91.8%, (**g**) 91.8% → 92.3%, (**h**) 92.3% → 92.8%.

**Figure 7:**
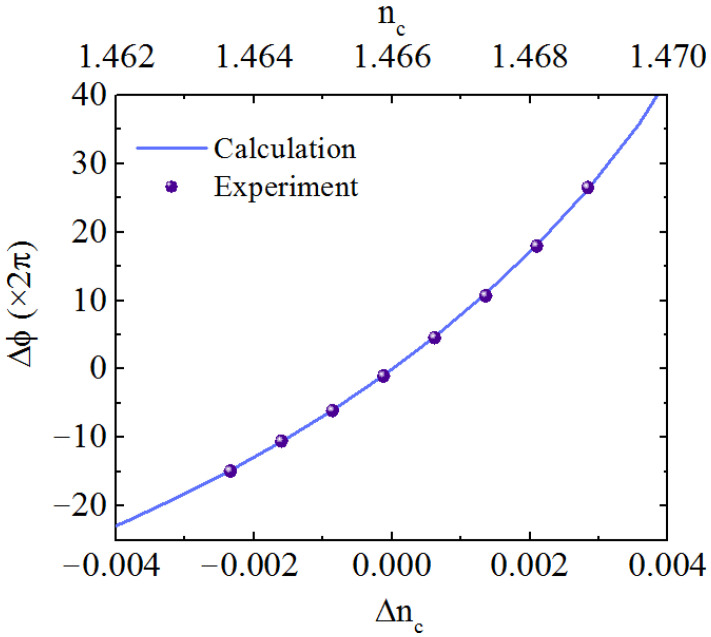
Phase change as a function of ∆*n_c_*; dots: experiment, solid line: calculation.

We used DMSO (*n*_DMSO_ = 1.480)/water solutions with different proportions as sensing analytes to obtain the MZI sensitivity. The refractive index of base DMSO/water solution was measured through the prism coupling method. The liquids were drawn into the sensing window through the PDMS channel by a syringe and different solutions were exchanged by moving the inlet of the Teflon tube into different beakers. When a DMSO/water solution with a higher refractive index flows into the sensing window to replace that with lower refractive index, an extra phase difference is caused, thus the output of MZI changes periodically as described by Equation (6), which leads to the corresponding oscillation of the intensity along with time. As the liquid switching process is finished, the output intensity becomes stable. Figure 6 shows the interferograms when DMSO/water solutions with DMSO volume percentage from 88.8% to 92.8% with a step of 0.5% (0.5% corresponds to 7.4 × 10^−4^ refractive index step estimating from the proportion of DMSO and water) passed through successively. The relative phase change was calculated from estimating the intensity variety of the output in Figure 6. As Equation (6) reveals, the intensity is a periodic function of the relative phase change and one sinusoidal period corresponds to 2π phase change. Then, relative phase changes of 7.44 π, 8.60 π, 9.06 π, 10.00 π, 11.06 π, 12.00 π, 13.92 π, 16.84 π were obtained, respectively. The higher the *n_c_*, the larger the relative phase change, indicating a higher waveguide sensitivity, which agrees with the conclusion derived from Figure 3b. The interference valley is close to zero, a high extinction ratio over 15 dB is measured of MZI. Figure 7 compares the experimental result of the phase variety ∆*ϕ* with the calculation (∆*n_c_* = *n_c_* − *n*_0_), they agree very well. The tangent slope of the curve in Figure 7 represents the MZI sensitivity as expressed by Equation (4). Thus the MZI sensitivities *S_d_* ranging from 10,556 π/RIU to 23,684 π/RIU were deduced. Taking MZI parameters *L* = 15 mm, λ = 632.8 nm, the sensitivity of 23,684 π/RIU corresponds to a waveguide sensitivity of *S_w_* = 0.50. In this case, the WCSW exhibited high waveguide sensitivity which was larger than silicon photonic wire waveguide which was *S_w_* = 0.35 [9] and polymer waveguide with *S_w_* < 0.1 [16]. We can see that the WCSW-based MZI sensor showed great superiority in sensitivity due to the large evanescent field distribution.

To further show the superiority of WCSW, the waveguide sensitivity in the case of symmetric mode field profile was calculated. When ∆*n_c_* = 0, the liquid has the same refractive index with cladding. The waveguide sensitivity *S_w_* of the WCSW is as large as 0.40 estimated from the fitting of experimental results, close to the waveguide sensitivity limit of 0.5. This proves that WCSW has high sensitivity even without adopting reverse symmetric waveguide.

**Figure 8:**
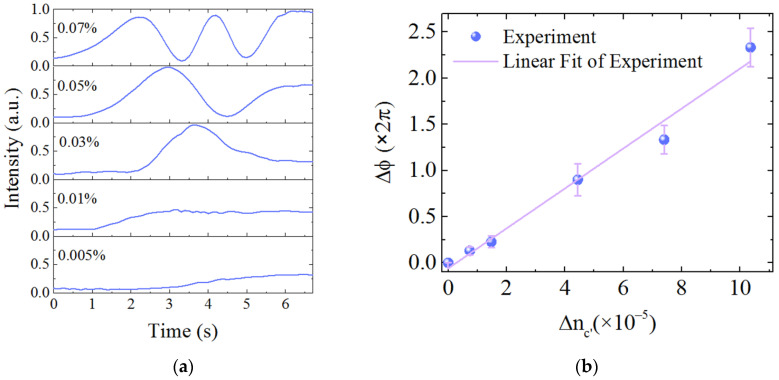
(**a**) Interference signals of DMSO volume ratio variety of 0.005%, 0.01%, 0.03%, 0.05%, 0.07%, respectively. (**b**) Linear fit of the experimental results with a slope of 44,364 π/RIU.

Near the mode cutoff area, the waveguide sensitivity can be greatly raised. We further tested the highest sensitivity of the WCSW based MZI sensor at higher *n_c_* with DMSO percentage of 94.5% (*n*_*c*__0_ = 1.472, taken as a base fluid). The results of DMSO volume percentage change of 0.07%, 0.05%, 0.03%, 0.01%, 0.005% in this refractive index region are plotted in Figure 8a (∆*n_c_*_’_ = *n_c_* − *n*_*c*__0_), corresponding to the ∆*n_c_*_’_ of 1.036 × 10^−4^, 7.40 × 10^−5^, 4.44 × 10^−5^, 1.48 × 10^−5^, 7.4 × 10^−6^, respectively. Phase changes of 4.80 π, 2.66 π, 1.76 π, 0.36 π, 0.25 π were obtained. Refractive index change of 7.4 × 10^−6^ (0.005% DMSO volume variety) was directly detected. Considering the sensitivity was nearly constant over a small range, the linear fitting of the experimental results with error bars was adopted and shown in Figure 8b. A sensitivity of *S_d_* = 44,364 π/RIU was obtained, which is almost 10 times higher than other MZI sensors [12,22,23,24]. A corresponding *S_w_* = 0.94 was deduced, which is very close to the waveguide sensitivity limit (*S_w_* = 1) in reverse symmetric waveguide. In this case, the waveguide mode field was largely distributed in the upper cladding as plotted in Figure 3c, and highly overlapped with liquids.

*LOD* of a sensor is defined by

(7) $$LOD=\frac{3\sigma }{S_{d}}$$

where *σ* is the signal standard deviation [12,23]. The phase resolution in our experiment is evaluated to be 3*σ* = 0.027 π by analyzing the noise level in Figure 8a and *LOD* as small as 6.1 × 10^−7^ RIU was obtained. The result is better than the measured *LOD* in [12,22,23,24,25,26] and close to the performance in [27,28,29], which is near the lowest *LOD* of MZI type sensor.

The WCSW has the advantage of high sensitivity but, in contrast, the sensing range is limited. This can be improved with proper waveguide parameter design. It is obvious that the sensitivity of WCSW is adjustable according to Figure 3b. The highest sensitivity is achieved in the mode near-cutoff area. By changing ∆*n* or *H*, the mode near-cutoff area of WCSW can sweep a large refractive index range. For higher refractive index liquids sensing such as benzene or toluene, the WCSW can provide high sensitivity as long as a higher ∆*n* or *H* is chosen, compared with sensing with DMSO/water solutions. Thus the WCSW is capable for large range high sensitivity detecting. On the other hand, when *n_c_* < *n*_0_, our new waveguide structure has no obvious advantages. However, preparing a low refractive index substrate material, such as porous silica, can allow the waveguide to work as a reverse symmetry waveguide, even in aqueous liquid, to achieve much higher sensitivity. Moreover, by increasing the sensing region length, a higher sensitivity can be achieved according to Equation (4). For example, spiral waveguides used in [30] showed the ability to raise sensing sensitivity. The Vernier effect based on cascaded ring and MZI [4] or two MZIs [24,31] could also provide a method for significant sensitivity improvement.

## 6. Conclusions

In summary, we have designed and fabricated an ultrasensitive Mach−Zehnder interferometer sensor based on a WCSW by sol−gel and DC-RTA techniques. The weak one-dimensional confinement of WCSW provides a large evanescent field in the vertical direction. Theoretical calculation shows that the proportion of evanescent field can reach 97%, the waveguide sensitivity can be very close to 1. The waveguide sensitivity is adjustable with proper waveguide parameter modulation, which provides a flexible choice when sensing with different liquids. Experimentally, we prepared WCSWs successfully and the waveguides were characterized with low propagation loss and low coupling loss with single-mode fiber. The refractive index sensing experiment with DMSO/water solutions were conducted. For symmetric waveguide, the waveguide sensitivity was measured to be 0.40, close to the limit of 0.5. For reverse symmetric waveguide, an ultrahigh waveguide sensitivity of 0.94, MZI sensitivity of 44,364 π/RIU and low *LOD* of 6.1 × 10^−7^ RIU were obtained, which is close to the best performance of MZI-type sensors.

## Data Availability

The data that support the findings of this study are available from the corresponding author upon reasonable request.
